# Dual Role of *Tenebrio molitor* Frass in Sustainable Agriculture: Effects on Free-Living Nematodes and Suppression of *Meloidogyne incognita*

**DOI:** 10.3390/biotech14030071

**Published:** 2025-09-08

**Authors:** Evgenia Rizou, Nikolaos Monokrousos, Triantafyllia Kardami, Georgia V. Baliota, Christos I. Rumbos, Christos G. Athanassiou, Nikolaos Tsiropoulos, Nikoletta Ntalli

**Affiliations:** 1 University Center of International Programmes of Studies, International Hellenic University, 57001 Thessaloniki, Greece; erizou@ihu.edu.gr; 2 Analytical Chemistry and Pesticides Laboratory, Department of Agriculture Crop Production and Rural Environment, University of Thessaly, 38446 Volos, Greece; tkardami@uth.gr (T.K.); ntsirop@uth.gr (N.T.); 3 Laboratory of Entomology and Agricultural Zoology, Department of Agriculture, Crop Production and Rural Environment, University of Thessaly, 38446 Volos, Greece; mpaliota@uth.gr (G.V.B.); athanassiou@uth.gr (C.G.A.); 4 Department of Agriculture, University of Patras, 30200 Messolonghi, Greece; crumbos@upatras.gr

**Keywords:** circular bioeconomy, nematicidal activity, network analysis, soil amendment, root knot nematodes

## Abstract

Insect-derived frass is gaining attention as a circular bioeconomy product with fertilizing and pest-suppressive potential. This study investigates *Tenebrio molitor* frass as a soil amendment for promoting beneficial nematodes and suppressing *Meloidogyne incognita*. A 40-day pot experiment on clay loam soil tested with six inputs: raw and heat-treated frass (0.5%, 1% *w*/*w*), *Melia azedarach* fruit powder (1.6%), and an untreated control. Soil nematode communities were assessed at 5 and 40 days after application (DAA), and nematicidal activity was evaluated in vitro. Raw frass at 1% induced a rapid response from free-living nematodes at 5 DAA, with increased abundance of bacterivorous taxa such as *Rhabditis* and *Acrobeloides*, alongside a higher Enrichment Index (EI), indicating short-term nutrient availability. At 40 DAA, only 1% raw frass consistently supported more cp-1 bacterivores and slightly increased Shannon diversity. Network analysis revealed more connected, modular structures in raw frass treatments, suggesting enhanced food web complexity. However, omnivore and predator effects were limited. Raw frass extracts caused over 80% paralysis of *Meloidogyne incognita* juveniles within 24 h, significantly outperforming heat-treated frass and *Melia* extracts. *T. molitor* frass moderately stimulates opportunistic nematodes and provides strong nematicidal effects, supporting its potential as a multifunctional input for sustainable soil management.

## 1. Introduction

Root Knot Nematodes (RKN) are phytoparasitic nematodes of the genus *Meloidogyne* sp. infesting all flowering plants and the most predominant species are *M. incognita* along with *M. arenaria*, *M. javanica*, and *M. hapla* [1,2]. The infective stage of the RKN is the second-stage juvenile (J2) which penetrates the root, typically just behind the tip. Then, the J2 migrates inside the root to establish a permanent feeding site. This feeding site consists of a few metabolically active multinucleate giant cells situated near the vascular bundles, supplying constant nourishment to the developing female. As a result, water and nutrient uptake is extensively reduced by the host plant followed by disease complexes involving other pathogens, like *Ralstonia solanacearum*, *Rhizoctonia solani*, *Fusarium oxysporum*, and *Thielaviopsis basicola* [3]. The presence of RKN in crops has become one of the major problems nowadays because they cause great agriculture loss. Among the many genera of nematodes having some economic impact, *Meloidogyne* spp. are responsible for a large part of the annual 100-billion-dollar losses attributed to nematode damage [4]. On the other hand, widespread soil degradation, intensive farming practices, and climate change are likely to accelerate the spread of soil nematodes [5]. The latter encompasses both phytoparasitic species, like the RKN, but also free-living species feeding on soil bacteria, fungi, and other nematodes, playing a vital role in litter decomposition, carbon, and nitrogen mineralization, adjusting the C:N ratio, nutrient recycling, carbon sequestration, and energy transfer [6,7,8,9]. These include a wide range of trophic groups—bacterivores, fungivores, omnivores, and predators—as well as plant-parasitic nematodes that spend at least one stage of their life cycle in the soil [10]. Their diversity and sensitivity to disturbance make them important components in evaluating the links between soil biodiversity, cropping systems, and ecosystem function [11].

Building on their central ecological role, nematodes have also been widely used as indicators of soil health and disturbance, thanks to their well-resolved trophic groups and colonizer–persister (c–p) classification [12]. Trait-based indices such as the Maturity Index (MI), Structure Index (SI), and Enrichment Index (EI) are frequently applied to assess food web complexity and soil health under different management regimes. MI reflects the level of environmental disturbance, SI indicates the complexity of the soil food web structure, and EI signals the availability of nutrients and resource enrichment [13]. More recently, co-occurrence network analysis has provided a complementary perspective by capturing shifts in community structure, trophic connectivity, and system robustness under environmental or anthropogenic pressures [14]. Despite increasing attention to organic soil amendments, including insect-derived frass, most studies have focused primarily on their suppressive effects against phytoparasitic nematodes, with limited attention to their broader impacts on non-target, free-living taxa. In this study, we explore how frass application affects both the composition and network structure of soil nematode communities, aiming to evaluate its dual role as a biological control input and a potential modulator of soil food web dynamics.

To date, adopted management strategies of the RKN focus on minimizing crop losses, enhancing ecosystem services, reducing greenhouse gas emissions, and improving the resilience of agricultural systems [5]. In this frame, the control of *Meloidogyne* sp. has been radically oriented to natural products, and most of the literature focuses on the use of botanicals [15,16], although many bioactive molecules might come from other natural resources. Τhe insect farming process generates as a waste product substantial amounts of insect frass of high potential for its biological properties. Specifically, frass has been shown in numerous studies to positively impact soil fertility [17,18], to produce pharmaceuticals, such as medicines and cosmetics, as well as biogas [19,20,21]. According to the Regulation (EU) 2021/1925, frass is “a mixture of excrements derived from farmed insects, the feeding substrate, parts of farmed insects, dead eggs and with a content of dead farmed insects of not more than 5% in volume and not more than 3% in weight” [22]. Frass composition varies highly depending on the insect species [23] as well as their feeding substrates [24]. Thus, different insect frass exhibit different properties. Two of the most studied frass are the ones deriving from the Black Soldier Fly (*Hermetia illucens*) and the mealworm (*Tenebrio molitor*).

In recent studies, the impact of olive pomace-derived Black Soldier Fly (*Hermetia illucens*) (Diptera: Stratiomyidae) frass (OP-BSF frass) was studied on soil health and plant growth by evaluating the soil model invertebrate *Enchytraeus crypticus* and the collembolan *Folsomia candida* and by conducting phytotoxicity bioassays using the forage crop ryegrass (*Lolium perenne*), broccoli (*Brassica oleracea*), onion (*Allium cepa*), turnip (*Brassica rapa*), and tomato (*Solanum lycopersicum*). No phytotoxicity was observed, the reproduction rate of *Enchytraeus crypticus* was augmented and intermediate rates of frass promoted plant development, thus implementing that OP-BSF frass could be incorporated into the EU Soil Strategy for 2030 so as to protect, restore, and promote the sustainable use of soils [25,26]. Indeed, the black soldier fly, *Hermetia illucens* (L.) has been since earlier times the subject of extensive research as an alternative to mineral fertilizers and synthetic pesticides in crop production [23,27]. Other studies have shown that black soldier fly larvae (*Hermetia illucens* L.), house crickets (*Acheta domesticus* L.), and yellow mealworms (*Tenebrio molitor* L.) frass have the potential to boost the activity of beneficial soil microorganisms [19,28].

On the other hand, frass from *T. molitor* in conjunction with an inorganic nitrogen fertilizer improved the growth, yield, and nutrient concentration in annual sow thistle plants [29]. But also used alone, frass from *T. molitor* has been proved an increaser of soil nutrient content, plant growth promoter, reducer of heavy metal content of the soil, increaser of flowers number [30], booster of soil carbon, and increaser of N uptake [31]. Lastly, bio-oil produced after pyrolysis of *T. molitor* frass showed bioinsecticidal effects against adult *Plodia interpunctella* and *Culex pipiens pipiens* larvae and exhibited low toxicity to the aquatic non-target species *Artemia salina* [32].

To establish a regulatory framework for frass production and its marketing as a biofertilizer, as well as standardizing its production across EU member states, the EU recently adopted Regulation 2021/1925 [22]. This regulation requires sanitizing before use, aligning frass with the standards applied to other processed animal manures. *Tenebrio molitor* (yellow mealworm/YMW) is currently allowed for frass production for food and feed [22] and to the best of our knowledge it has yet to be studied for its nematicidal activities against *Meloidogyne incognita* and its secondary effects on soil free-living nematodes.

Given the increasing interest in sustainable, multifunctional soil amendments, this study aims primarily to investigate how *T. molitor* frass influences the structure, trophic composition, and interaction networks of soil free-living nematodes—organisms central to nutrient cycling and ecological resilience. We assessed nematode community responses using trait-based indices and co-occurrence network analysis to evaluate whether frass application alters food web structure, enriches opportunistic taxa, or modifies ecosystem stability. In addition, we present preliminary findings on the nematicidal activity of frass against *Meloidogyne incognita*. Most interestingly, we study all the above by comparing effects of untreated and sanitized frass (heated at 70 °C for 1 h) to evaluate the effect of sanitation on this organic matrix. This microcosm study provides an initial assessment of *T. molitor* frass as a soil amendment, focusing on free-living nematode communities and in vitro nematicidal activity. While the findings offer insights into potential mechanisms and effects, further research under field or mesocosm conditions, including plant hosts and longer time frames, is needed to fully evaluate agronomic impacts.

## 2. Materials and Methods

### 2.1. Insect Rearing and Frass Collection

The frass produced by *T. molitor* larvae utilized in the present study was obtained from the pilot insect rearing facility of the Laboratory of Entomology and Agricultural Zoology, University of Thessaly, Greece. Larvae were reared in plastic containers (trays) (60 × 40 × 14.5 cm; Beekenkamp Verpakkingen BV, Maasdijk, The Netherlands) under controlled environmental conditions (27 ± 0.5 °C; 60 ± 5% relative humidity) and complete darkness. Wheat bran was provided as a dry feed substrate, while agar (20 g/L) was supplied three times per week as a moisture source. Upon feed depletion and the initiation of pupation is approximately 10% of the larval population, frass was separated from the larvae by mechanical sieving through a 500 μm mesh.

For the purposes of the study, two distinct frass batches were prepared: (i) frass collected directly from the rearing trays without any post-collection treatment, and (ii) frass subjected to thermal treatment at 70 °C for 1 h, following the protocol described by Karkanis et al. [29]. The physiochemical properties of frass were pH: 5.5, organic matter: 90%, total nitrogen: 34 mg/kg, nitrate nitrogen: 103 mg/kg, phosphorus: 3813 mg/kg, and potassium: 22 mg/kg. After treatment, the frass was allowed to cool to ambient temperature and subsequently stored in plastic containers under room conditions until further analytical and experimental use.

### 2.2. Experimental Design and Justification

This experiment was designed as a controlled microcosm study to isolate and assess the direct effects of *T. molitor* frass on soil free-living nematode communities and nematicidal activity. Host plants were intentionally excluded to focus on the mechanistic responses of soil nematodes to frass amendments without the confounding influence of plant–nematode interactions. This approach was chosen due to resource constraints and to serve as a preliminary step before more complex, plant-based or field assays.

Only two sampling points (5 and 40 days after application, DAA) and two frass concentrations (0.5% and 1% *w*/*w*) were selected. This limited design reflects the pilot nature of the study, aiming to capture both immediate and short-term responses while balancing feasibility and resource availability. The chosen concentrations represent realistic amendment rates based on prior studies and regulatory guidance [31].

### 2.3. Free-Living Nematodes in Soil Bioassays

Clay loam soil with 2.4% content of organic matter, pH of 7.7, EC of 2.4% and total P of 138.5% was collected from an uncultivated field at the University Farm. The soil was passed through a 3 mm sieve to remove debris and then was partially air-dried overnight. The soil was divided into 12 plastic bags, corresponding to six treatments (1.6 kg per bag) and two time intervals, that is at 5 and 40 DAA (Days After Application). The experimental treatments were heated frass at 0.5%, heated frass at 1%, raw frass at 0.5%, raw frass at 1%, water (negative control), and *Melia azedarach* fruit powder at 1.6% (positive control). We chose MFP as a positive control for our experiment because, similarly to frass, it is organic and we already proved its activity on *Meloidogyne incognita*, 70% efficacy according to Ntalli et al. [33], and its beneficial effect on soil free-living nematodes in previous studies. The fortification of the soil was performed by mixing frass powder with the soil and passing through a 3 mm sieve twice. Each bag was then divided into 10 pots, watered with 10 mL every 3 days, and kept under 27 °C, 60% relative humidity, with a 16 h photoperiod.

### 2.4. Nematode Extraction

Nematodes were extracted from 100 g of each soil sample, after undergoing a natural degradation of soil aggregates. Nematode extraction was performed using Cobb’s sieving and decanting technique, as modified by S’Jacob and van Bezooijen [34], which includes the use of a cotton wool filter at the end of the process. After estimating the total nematode abundance using a stereoscope (Euromex model NZ-1703-PGA, Typograaf 8 6921 VB Duiven, The Netherlands), they were preserved in 4% formaldehyde solution using a heat-killing and fixing procedure [11]. Subsequent to preservation, 100 nematodes were randomly selected from each sample and taxonomically identified at the genus level using Bongers’ identification key [35], supplemented and cross-referenced with the Nemaplex online database to ensure updated and accurate taxonomic assignments. In accordance with the standards established by Yeates et al. [36], the nematode genera were divided into trophic groups and arranged along the colonization–persistence gradient (c-p values) [37,38]. Regarding nematode functional indices, the Plant-Parasitic Index (PPI) measures the abundance and maturity of plant-parasitic nematode communities and their effect on plant health. The Maturity Index (MI) measures the successional status and disturbance level of free-living nematode communities, with increasing values pointing to more stable, undisturbed ecosystems. The Structure Index (SI) measures the complexity and connectedness of the soil food web, and the Enrichment Index (EI) measures nutrient enrichment and availability of resources triggering opportunistic nematodes. The Channel Index (CI) indicates the prevailing decomposition channel, separating bacterial and fungal decomposition processes. Together, these indices give insight into soil ecosystem dynamics and health [37,38,39].

### 2.5. Meloidogyne incognita Rearing

A population of *M. incognita*, initially obtained from naturally infested tomato roots, was propagated on tomato (*Solanum lycopersicum* L. cv. Belladonna), a cultivar highly susceptible to root knot nematodes. The plants were grown in a controlled environment chamber at 25–28 °C, 60% relative humidity, and a 16 h photoperiod. They were cultivated in plastic pots (18 cm in diameter) filled with peat. Inoculation was performed at the five-leaf stage. After 30 days, the plants were uprooted, and their roots were washed to remove soil, then cut into 2 cm pieces. Egg extraction was carried out using sodium hypochlorite, according to Hussey and Barker [40]. Second-stage juveniles (J2) were hatched in modified Baermann funnels at 28 °C, with those emerging in the first three days discarded, while J2 collected in the following 24 h were used for the experiments.

### 2.6. Meloidogyne incognita Paralysis Bioassays on the Effect of Water Extract of Collected Frass from Tenebrio molitor

*Tenebrio molitor* frass was divided into two parts. The first half was used directly for the extraction with water to furnish frass water extract, while the second part was first sanitized, that is heated at 70 °C for one hour, and later extracted with water to give heated frass water extract. The extraction was performed with water (1:10 *w*/*v*), sonication for 15 min, and filtered through cotton. Both extracts were tested on *M. incognita* at the test concentration range of 1–50% (*v*/*v*), for EC_50_ values calculation. The bioassays were conducted in Cellstar 96-well cell culture plates (Greiner Bio-One, Kremsmünster, Austria), with each treatment encompassing 25 J2 per well. The plates were protected with plastic lids and kept in the dark at 28 °C. Juvenile nematodes were observed at 24, 48, and 72 h using an inverted microscope (Euromex, Arnhem, The Netherlands) at 40× magnification and categorized into two groups: motile or paralyzed. The paralysis experiments were performed twice, and every treatment was replicated six times.

### 2.7. Statistical Analysis

To examine the effects of frass application on soil nematode communities, a combination of univariate and multivariate statistical approaches was employed. A two-way ANOVA was used to test the effects of the treatments and time after application, as well as their interaction with soil nematode abundance and nematode functional indices. Post hoc comparisons were performed using Tukey’s Honest Significant Difference (HSD) test at a significance threshold of *p* < 0.05. Prior to analysis, the assumptions of ANOVA (normality wand homogeneity of variance) were checked; when necessary, data were appropriately log-transformed to meet these assumptions before analysis. All analyses were conducted with Statistica 7 (StatSoft, Tusla, OK, USA).

To evaluate shifts in nematode community composition, non-metric multidimensional scaling (NMDS) and SIMPER analyses were conducted based on Bray–Curtis dissimilarities. NMDS was used to visualize structural differences among treatments in response to frass amendments. Additionally, Rényi diversity profiles [41] were calculated following the method of Patil and Taillie [42], allowing sensitivity to both rare and dominant taxa by varying the diversity order (α). All computations were performed in PAST (version 4.03). To further explore treatment effects on community structure, network analysis was applied, where nodes represent nematode genera and edges indicate their co-occurrence across samples. Community matrices were constructed separately for each treatment and analyzed using UCINET 6. Metrics describing density, compactness, and fragmentation of the network structure were calculated to evaluate overall network cohesion. Additional methodological details are available in Dimou et al. [16].

Regarding the root knot nematodes, paralysis data were replicated six times, and each experiment was conducted twice. The percentage of paralyzed J2 in the microwell assays was adjusted to account for natural death/paralysis in the water control using the Schneider-Orelli formula:

Corrected % = [100 − Mortality % in control Mortality % in treatment − Mortality % in control] × 100

Data were analyzed using ANOVA across time points. Since ANOVA showed no significant interaction between treatment and time, means were averaged across experiments. Corrected percentages of paralyzed J2 exposed to the test substance(s) were analyzed using nonlinear regression based on the log-logistic equation proposed by Seefeldt et al. [43]:

Y = C + 1 + exp[*b*(log(x) − log(EC_50_)](D − C)

where C represents the lower limit, D represents the upper limit, *b* is the slope at EC_50_., and EC_50_ is the concentration of the test substance required to induce 50% paralysis or death after correcting for natural mortality. In the regression equation, the concentration of the test substance (% *v*/*v*) was the independent variable (x), while the percentage increase in paralyzed J2 compared to the water control was the dependent variable (y). The mean value from five replicates per test concentration and immersion period was used to calculate the EC_50_ value.

## 3. Results

Nematodes from all trophic groups were recorded across all five treatments and the control. Specifically, bacterivores belonged to the genera *Acrobeles*, *Acrobeloides*, *Chiloplacus*, *Eucephalobus*, *Heterocephalobus*, *Mesorhabditis*, *Monhystera*, *Panagrolaimus*, and *Rhabditis*; fungivores to *Aphelenchoides* and *Aphelenchus*; herbivores included *Bitylenchus* (ectoparasitic), *Helicotylenchus* (semi-endoparasitic), and *Meloidogyne* (sedentary endoparasitic) genera; and omnivores were represented by the genus *Dorydorella* (Table 1). No predators were found.

**Figure 1:**
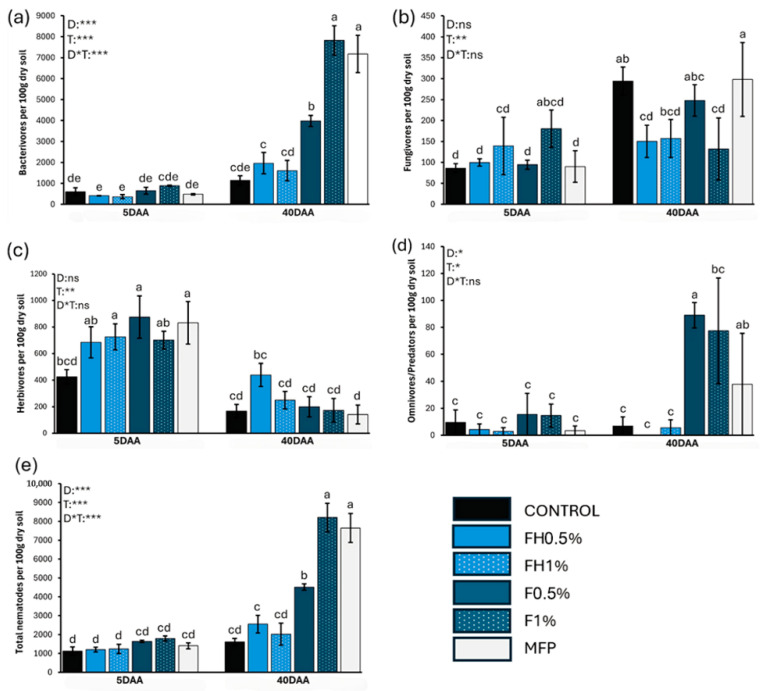
(**a**–**e**). Mean abundance (±standard error) of nematode trophic groups [(**a**) bacterivores, (**b**) fungivores, (**c**) herbivores, (**d**) omnivores/predators, and (**e**) total nematode abundance] across treatments, along with results from two-way ANOVA assessing the effects of Time (T), Treatment (D), and their interaction (D × T) at two sampling points (5 and 40 days after application, DAA). Different letters above bars denote statistically significant differences among treatments according to Tukey’s post hoc test (*: *p* < 0.05; **: *p* < 0.01; ***: *p* < 0.001; ns: not significant). Sample size *n* = 4 in all cases.

**Figure 2:**
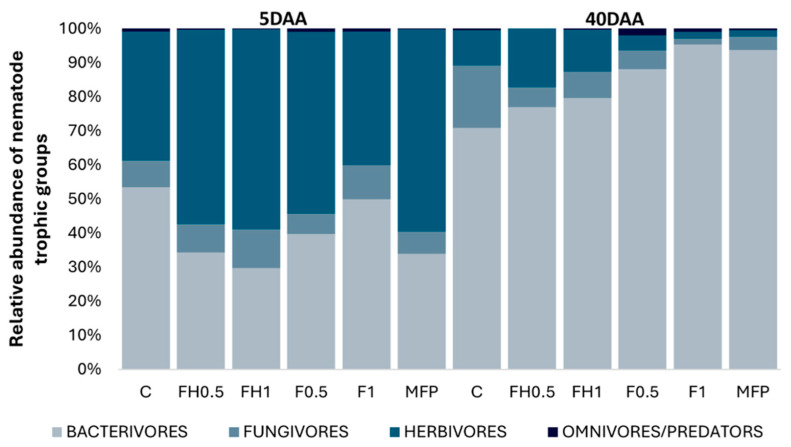
Relative abundance (%) of different nematode trophic groups after the application of different frass treatments at 5 and 40 DAA (Days After Application). Different colors indicate the different trophic groups and abbreviations indicate the six different treatments (C: control; FH0.5: heated frass in 0.5% dose; FH1: heated frass in 1% dose; F0.5: raw frass in 0.5% dose; F1: raw frass in 1% dose; MFP: *M. azedarach* fruit powder).

Figure 1a–e illustrates the abundance of nematode trophic groups across treatments at 5 and 40 days after the application of frass (DAA). A significant effect of the sampling period was observed (*p* < 0.01), primarily due to the higher abundances of bacterivores and omnivores in the naturally decomposed frass (F) treatments at 40 DAA. However, no statistically significant differences were found over time for the other free-living nematode groups. Bacterivores represented the most abundant trophic group, followed by herbivores, fungivores, and omnivores/predators (Figure 1 and Figure 2). Bacterivore populations increased notably over time, particularly in the F0.5, F1, and MFP treatments, suggesting elevated microbial activity. In contrast, fungivore abundance remained low at 5 DAA, with no clear treatment-dependent trends.

A moderate increase in fungivore abundance was observed at 40 DAA, with the control (C) exhibiting the highest values compared to the other treatments (Figure 1). Although the effects of treatment and the interaction between time and treatment (D × T) were not statistically significant, time had a significant effect (*p* < 0.01), indicating that temporal dynamics played a greater role in shaping fungivore populations than treatment type. This is likely due to the delayed development of fungal biomass, which serves as a food source for fungivorous nematodes. Herbivore populations at 5 DAA increased in FH1, F0.5, and MFP treatments relative to the control, but their numbers declined markedly in most treatments by 40 DAA. While D and D × T effects were again not significant, time exerted a strong influence (*p* < 0.01), suggesting that temporal factors were primarily responsible for the observed population shifts. At 5 DAA, no clear differences among treatments were found in the abundance of omnivores and predators. However, their numbers increased by 40 DAA, particularly in the F0.5 and F1 treatments, which may reflect emerging trophic stabilization and restructuring of soil food web dynamics. Overall, nematode populations increased significantly over time. Total nematode abundance was relatively low and uniform across treatments at 5 DAA, but increased sharply by 40 DAA, with F0.5, F1, and MFP treatments exhibiting substantially higher densities than the others (Figure 1).

The strong influence of both treatment and time on overall nematode abundance was confirmed by statistical analysis, which revealed highly significant effects of treatment (D; *p* < 0.001), time (T; *p* < 0.001), and their interaction (D × T; *p* < 0.001).

Figure 2 illustrates changes in the relative abundance of nematode trophic groups over time. At 5 DAA, community composition was relatively balanced across all treatments, with herbivores, fungivores, bacterivores, and omnivores/predators each contributing comparably. During this early stage, herbivores and bacterivores displayed the highest relative abundances. However, by 40 DAA, herbivore representation had significantly declined, and bacterivores became the dominant trophic group in all treatments, indicating a marked shift in community composition. Omnivores and predators showed a modest increase in certain treatments, particularly F0.5 and F1, while fungivores and herbivores remained low overall. A slight increase in fungivore abundance in the control at 40 DAA suggests some variation in fungal-associated nematode dynamics.

**Figure 3:**
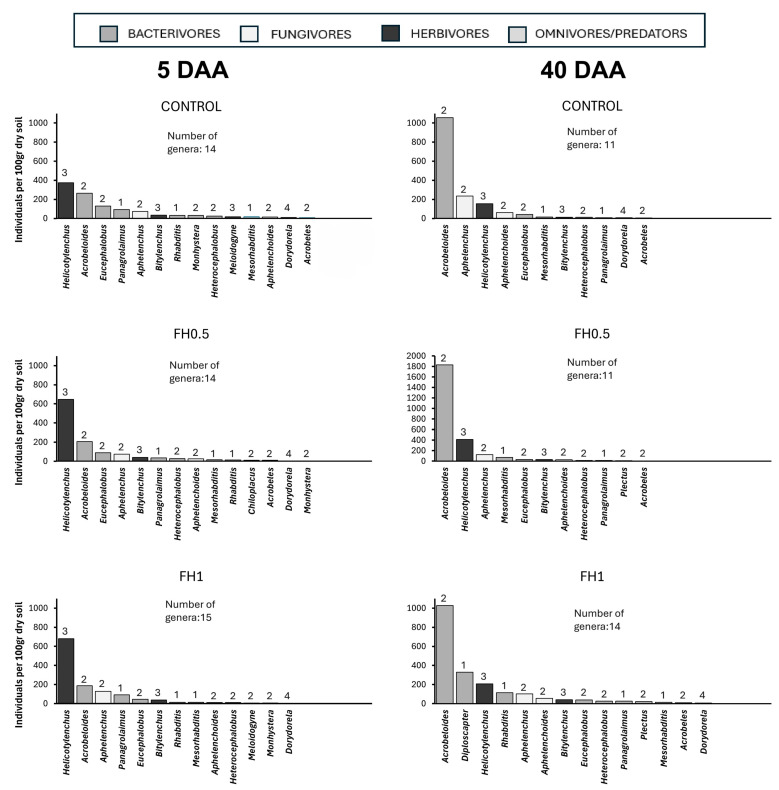

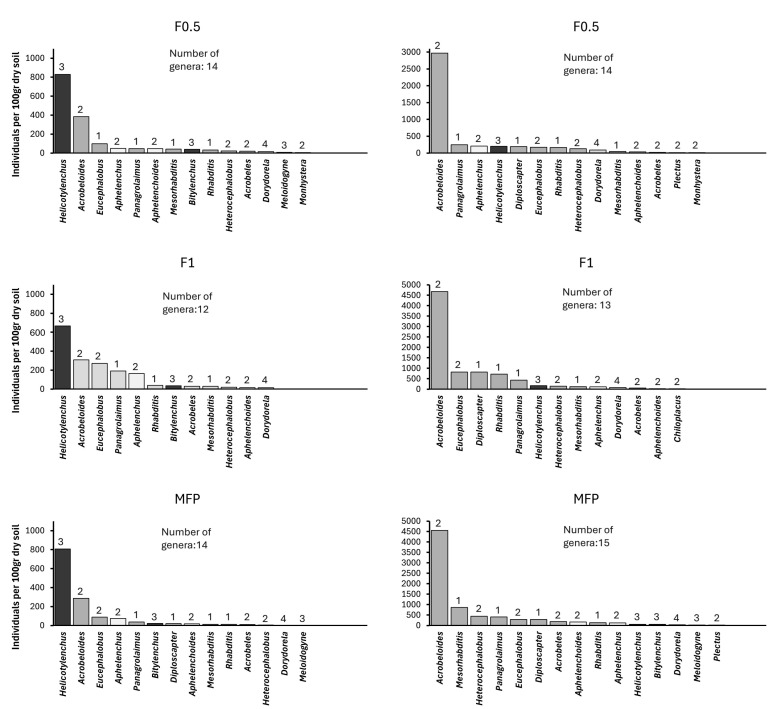
Rank-abundance curves showing the distribution of nematode genera across different treatments at 5 and 40 days after application (first and second column, respectively), including control, heated frass at 0.5% (FH0.5) and 1% (FH1), raw frass at 0.5% (F0.5) and 1% (F1), and *Melia azedarach* fruit powder (MFP). Genera are ranked from most to least abundant, with the c-p value indicated above each bar. Colors denote the trophic group of each genus. Sample size was *n* = 4 for all treatments.

The rank-abundance graphs indicated that nematode populations in the control samples (C5 and C40) exhibited a more balanced distribution of genera and remained relatively stable between 5 and 40 DAA (Figure 3). The persistence of genera from higher colonizer–persister (c–p) categories, such as *Heterocephalobus*, *Mesorhabditis*, and members of the *Dorylaimida*, over time suggests a more structured and less disturbed soil environment. In contrast, at 5 DAA, treated samples (FH, F, and MFP) showed a marked predominance of genera such as *Rhabditis*, *Diploscapter*, and *Aphelenchus*, indicative of an initial enrichment phase driven by organic inputs. By 40 DAA, nematode community composition had shifted, with changes in the persistence or decline of dominant genera depending on the specific treatment (Figure 3). The notable increase in bacterivorous genera such as *Acrobeloides*, *Rhabditis*, and *Diploscapter*, particularly in the F and MFP treatments, points to prolonged habitat enrichment or disturbance linked to frass application. At 5 DAA, the nematode communities across treatments were largely composed of r-strategists (cp-1 and cp-2 taxa). In some treatments, the relative abundance of these colonizers declined by 40 DAA (e.g., *Rhabditis*, *Acrobeloides*), suggesting progressive stabilization of the soil environment. In other treatments, however, their continued dominance indicated persistent disturbance. The presence and persistence of higher c–p value nematodes (e.g., *Heterocephalobus*, *Dorylaimida*) under specific treatments point to the beginning of a recovery process toward equilibrium, whereas the continued dominance of opportunistic genera in other treatments suggests ongoing environmental stress (e.g., continued high availability of labile organic inputs from frass amendments, which sustains the dominance of opportunistic colonizer nematodes). Treatment-specific effects became more pronounced over time. At 5 DAA, early-stage colonizers increased substantially in response to amendments such as F and MFP (*p* < 0.01). By 40 DAA, community composition diverged depending on treatment. For instance, *Acrobeloides* and *Helicotylenchus* remained dominant in the FH treatments, possibly reflecting either long-term disruption or adaptation to altered conditions. The most notable shift occurred under the MFP treatment, where *Acrobeloides* and *Mesorhabditis* increased significantly by day 40 (*p* < 0.01), suggesting continued microbial stimulation or signs of sustained ecosystem stress. Overall, while the control samples maintained a relatively balanced nematode assemblage over time, the experimental treatments favored the proliferation of opportunistic decomposers, especially during the early stages of decomposition.

**Figure 4:**
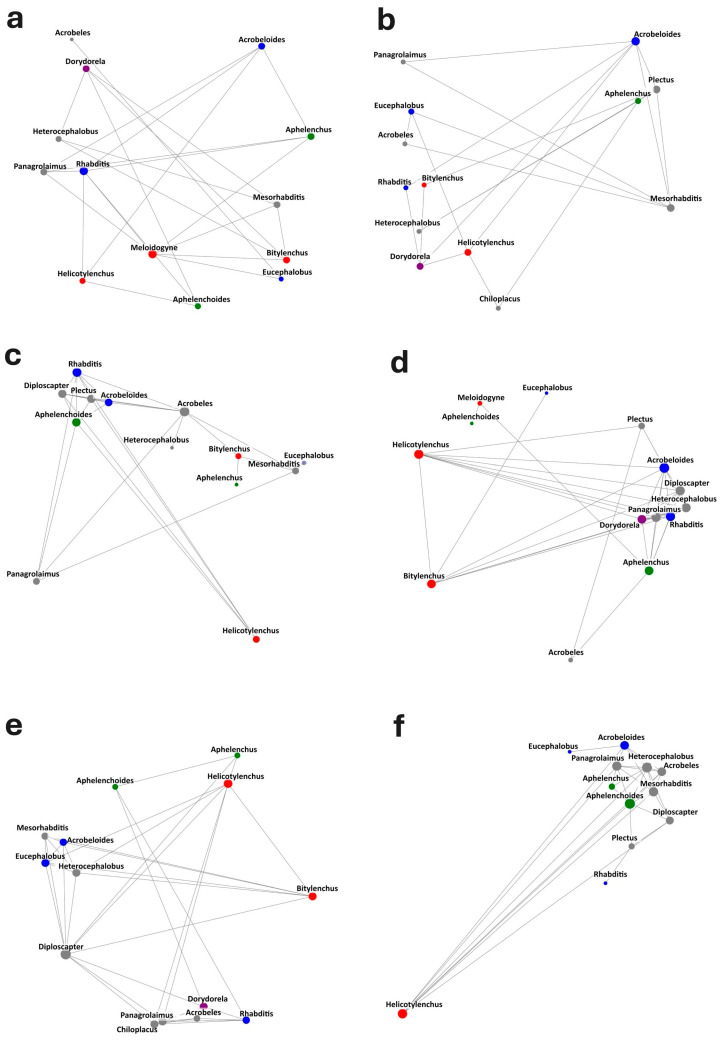
(**a**–**f**) Co-occurrence networks of nematode genera across different treatments visualized 40 days post-application. Treatments include: (**a**) control, (**b**) heated frass at 0.5% dose, (**c**) heated frass at 1% dose, (**d**) raw frass at 0.5% dose, (**e**) raw frass at 1% dose, and (**f**) *Melia azedarach* fruit powder. Node size reflects the degree of connectivity (i.e., the number of co-occurrences with other genera). Node color indicates the trophic group affiliation of each nematode genus (Gray: bacterivores; red: plant-parasitic; green: fungivores; purple: omnivores).

According to the co-occurrence networks shown in Figure 4a–f, the F0.5 and F1 treatments exhibited more cohesive nematode networks, whereas the FH0.5 network appeared more fragmented and less connected. These observations are supported by the network metrics presented in Table 2. The FH0.5 treatment showed the lowest number of edges (21), lowest average degree (3.231), and lowest network density (0.269), indicating weaker interactions among nematode genera. Additionally, its average clustering coefficient (0.456) was among the lowest, suggesting reduced local interconnectedness. In contrast, the F0.5 and F1 treatments had higher edge counts (40 and 39, respectively), greater average degrees (5.714 and 5.571), and higher densities (0.440 and 0.429), reflecting stronger co-occurrence and greater ecological complexity within these communities. Notably, the F1 network also had the highest clustering coefficient (0.700), indicating a more structured and tightly connected community. These patterns suggest that certain treatments—particularly those involving unheated frass—may enhance trophic interactions and microbial stability, likely due to a more favorable substrate composition and nutrient availability.

**Figure 5:**
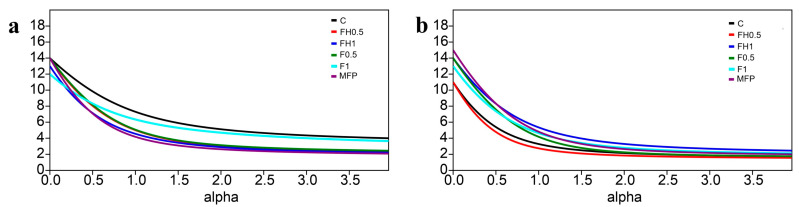
(**a**,**b**) Diversity profiles of nematode communities in the six treatments (C: control; FH0.5: heated frass in 0.5% dose; FH1: heated frass in 1% dose; F0.5: raw frass in 0.5% dose; F1: raw frass in 1% dose; and MFP: *M. azedarach* fruit powder at (**a**) 5 and (**b**) 40 days after application. When the parameter α is set to 0, 1, and 2, Renyi’s diversity index corresponds to species richness (number of genera), the Shannon index, and the Simpson index, respectively.

The Rényi diversity profiles revealed that treated samples maintained relatively higher diversity at α = 1 and α = 2, while species richness (α = 0) declined over time, particularly in the control group (Figure 5). Initially, diversity values at α = 0 were higher at 5 DAA than at 40 DAA, reflecting greater species richness during early stages, especially in the control. However, richness in treated samples declined gradually over time. At α = 1, which accounts for both richness and evenness, diversity also declined, suggesting the emergence of dominant species. The control group showed a more pronounced decline in diversity at 40 DAA, indicating a shift in community structure in the absence of inputs. At α = 2, where dominant taxa are weighted more heavily, diversity continued to decrease, implying that certain MFP (microbivorous, fungivorous, or plant-parasitic) species became dominant over time. In contrast, frass-treated samples often retained higher diversity at 40 DAA, suggesting that frass contributed to maintaining a more balanced nematode community structure.

The Structure Index (SI) values did not differ significantly across sampling times, treatments, or their interaction (Table 3). Similarly, the Plant-Parasitic Index (PPI) remained stable, with no significant changes detected. Maturity Index (MI) values remained relatively consistent across treatments at both 5 and 40 days after application (DAA), with the highest values observed in F0.5 (1.91 ± 0.10) at 5 DAA and FH0.5 (1.96 ± 0.01) at 40 DAA. The lowest MI values were recorded in F1 (1.75 ± 0.10) and FH1 (1.75 ± 0.11) at 40 DAA. In contrast, Enrichment Index (EI) values showed marked variability. At 5 DAA, the highest EI values were observed in FH1 (59.51 ± 3.70) and F1 (59.10 ± 4.32), while the lowest value occurred in FH0.5 (19.17 ± 2.64) at 40 DAA. Although SI values varied numerically across treatments and times, no statistically significant differences were detected. The Channel Index (CI) was uniquely affected by the combined influence of sampling time and treatment, with its highest value observed in the control group at 40 days after application (77.31 ± 9.35), whereas the lowest CI value was observed in MFP at 40 DAA (4.40 ± 1.36), suggesting a shift in decomposition pathways.

**Figure 6:**
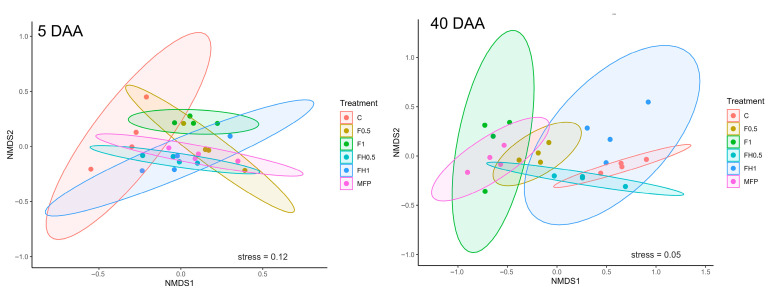
Ordination of nematode communities using non-metric multidimensional scaling (NMDS) based on Bray–Curtis dissimilarity calculated from relative abundances of nematode genera. Samples were taken at 5 and 40 days following frass application (DAA). Each point represents the nematode community from an individual sample (*n* = 4 per treatment). Ellipses represent the 95% confidence area for each treatment group. NMDS was performed using Bray–Curtis dissimilarities with k = 2 dimensions; stress values were 0.12 (5DAA) and 0.05 (40DAA), indicating an adequate fit. The presence of overlapping groups implies that certain treatments had similar initial effects on nematode composition (C: control; FH0.5: heated frass in 0.5% dose; FH1: heated frass in 1% dose; F0.5: raw frass in 0.5% dose; F1: raw frass in 1% dose; MFP: *M. azedarach* fruit powder).

The NMDS ordination plots (Figure 6) illustrated temporal changes in nematode community composition in response to the treatments. At 5 DAA, nematode communities exhibited high variability, with more dispersed groupings and overlapping clusters, indicating an early and dynamic response to the applied treatments. The control treatment showed marked separation from treated samples, suggesting a distinct baseline community structure. By 40 DAA, communities appeared more stable, and treatment effects became more defined. Each group formed tighter clusters, with FH1 positioned apart from the others in ordination space, implying that this treatment exerted a strong and lasting influence on nematode community composition (Table S1). This pattern suggests that early community shifts were rapid and variable, while longer-term responses led to more distinct, treatment-driven assemblages.

As far as the efficacy of *T. molitor* frass on *M. incognita* second-stage juveniles is concerned, the non-sanitized frass was more active, and this activity was stable in time (Table 4). In particular, the EC_50_ value was calculated at 10% *v*/*v* for juveniles immersed in test solutions for 24 h. This effect remained stable throughout the whole bioassay duration and since J2 never regained motility up until 96 h post bioassay establishment they were characterized as dead. On the other hand, the heated frass gave an EC_50_ value of 13.84% *v*/*v* at the first assessment date (24 h) but to some extent, during the following hours, some J2 regained their motility, and the paralysis effect stabilized 48 h post experiment establishment with the EC_50_ value calculated at 20% *v*/*v*. At the final assessment date, the EC_50_ value was again calculated at 20% *v*/*v* and the paralyzed J2s never regained their motility and were characterized as dead (Table 4).

## 4. Discussion

In our study, the free-living nematode community was predominantly composed of microbial feeders, particularly bacterivores, with notable shifts induced by the frass treatments. Omnivores and predators, which are highly sensitive to soil disturbance [40], were recorded in low numbers throughout the experimental period and appeared primarily at 40 DAA. The abundance of plant-parasitic nematodes remained low across both sampling periods, likely due to the absence of host plants in the experimental setup, which prevented the establishment of a stable herbivore population [44].

Frass amendments led to substantial changes in both the trophic structure and taxonomic composition of the nematode community. Bacterivorous nematodes increased significantly in the F0.5, F1, and MFP treatments, with cp-1 and cp-2 taxa such as *Acrobeles*, *Acrobeloides*, and *Rhabditis* dominating the communities at 40 DAA (*p* < 0.01). This is consistent with previous findings showing that organic inputs stimulate microbial activity, thereby favoring bacterial-feeding nematodes [45]. Similarly to observations in *Melia azedarach* applications [46], frass likely contributed organic substrates that promoted bacterial proliferation, leading to enhanced populations of bacterivores [47]. The marked increase in cp-1 bacterivores, particularly *Rhabditis*, suggests rapid bacterial blooms following amendment, as these enrichment opportunists are known for their short life cycles and high reproductive rates [6]. This pattern is reflected in the elevated Enrichment Index (EI) and reduced Channel Index (CI) values observed in frass-amended treatments, indicating a dominance of bacterial over fungal decomposition pathways.

Despite the increase in bacterivores, fungivorous nematodes (*Aphelenchoides*, *Aphelenchus*) showed a moderate rise at 40 DAA, with the highest abundance recorded in the control treatment. The Channel Index (CI) supported this observation, indicating that only the control at 40 DAA had CI values exceeding 50%, suggesting fungal decomposition was the dominant pathway [40]. The persistence of fungal-feeding nematodes in the control likely reflects the continued availability of undecomposed crop residues, which provided a stable energy source for fungal growth [48]. According to Figure 1b, although fungivore populations in the control showed signs of increasing, frass-treated samples did not support similarly high abundances. This may be attributed to the presence of chitin and other bioactive compounds in frass, which can suppress fungal development and potentially inhibit the growth or reproduction of fungivores, particularly at juvenile stages [49].

Herbivorous nematodes (*Bitylenchus*, *Helicotylenchus*, *Meloidogyne*) exhibited an initial increase at 5 DAA, particularly in FH1, F0.5, and MFP treatments, but their populations declined markedly by 40 DAA. Statistical analysis confirmed that time (T; *p* < 0.01) was the principal factor influencing this decline. Similar reductions in herbivore abundance have been reported in studies applying botanical nematicides, which may indirectly affect herbivores by altering soil physicochemical conditions or microbial interactions [45]. Furthermore, the absence of host plants in the experimental setup likely limited the persistence of these plant-parasitic species [44].

Omnivores and predators, represented primarily by *Dorydorella*, showed no significant treatment differences at 5 DAA. However, by 40 DAA their abundance increased in the F0.5 and F1 treatments, indicating a trend toward trophic stabilization. The increased presence of cp-4 omnivores and predators suggests improvement in soil biological condition, as these taxa play a critical role in regulating bacterial and fungal feeders. contributing to nutrient cycling and soil food web complexity [50,51]. These observations are consistent with prior findings that organic amendments promote more structured and resilient nematode communities over time. The absence of a significant increase in omnivores and predators, and the limited improvement in indices such as the Structure Index (SI), suggest that the overall food web complexity and functional diversity were not markedly enhanced within the timeframe of this microcosm experiment.

Total nematode abundance was relatively uniform across treatments at 5 DAA but increased significantly in the F0.5, F1, and MFP treatments by 40 DAA. Statistical analysis confirmed significant effects of treatment, time, and their interaction (*p* < 0.001), reflecting the combined influence of organic inputs and time-dependent ecological processes. These results are in line with previous studies demonstrating that organic amendments support the proliferation of free-living nematodes more effectively than chemical treatments [46,52]. Regarding plant productivity, increased bacterivorous nematodes after frass amendments indicate enhanced bacterial activity and nutrient cycling, which generally benefits soil fertility and crop growth. The low abundance of fungivores and herbivores suggests a shift in decomposition pathways and reduced plant-parasitic pressure, respectively. The modest rise in omnivores and predators points to gradual trophic stabilization. Overall, these changes imply a positive impact on soil health and sustainable crop production, though longer-term monitoring of food web complexity is recommended. We acknowledge that the 40-day duration of this study limits assessment of long-term effects of frass on soil ecosystems. However, this timeframe is sufficient to capture key initial responses and recovery phases of free-living nematode communities. The exclusion of plants was intentional to isolate direct effects of frass amendments on soil fauna without confounding plant–nematode interactions. Future work incorporating longer durations and living plants will be valuable to fully understand agronomic impacts.

Diversity profiles indicated that species richness (α = 0) declined over time, particularly in the control (C), despite initially higher values at 5 DAA. By 40 DAA, however, frass-treated samples maintained a more balanced nematode community composition, avoiding the dominance of individual taxa. This trend aligns with the ecological effects of botanical amendments, which are known to promote soil biodiversity and enhance trophic interactions. These findings are consistent with previous research demonstrating that organic inputs contribute to the development of more complex and resilient soil food webs [53]. A well-structured food web is critical for sustaining essential ecosystem functions such as nutrient retention, organic matter decomposition, and soil structural stability. Nevertheless, the dominance of bacterivores and the scarcity of higher c–p value nematodes highlight the limited trophic structure and resilience of the system under the tested conditions. In contrast, soils treated with chemical amendments often exhibit fragmented networks and bottom-heavy structures, which can impede predator mobility and disrupt trophic linkages [54].

Network analysis revealed distinct structural differences among nematode communities across treatments. Among treatments, FH0.5 resulted in the highest degree of network fragmentation, with the lowest number of edges, average degree, and clustering coefficient, indicating a less interactive and potentially more disturbed community. In contrast, the F0.5 and F1 treatments displayed highly connected networks, with greater edge counts, higher network density, and stronger clustering, suggesting robust interactions among genera. These findings are consistent with previous studies showing that organic inputs can enhance soil biological networks, promoting trophic complexity and stability [55]. Enhanced clustering and connectivity are often associated with increased resource availability and microbial activity, which support the coexistence of diverse functional groups [56]. On the other hand, fragmented networks, such as those observed under chemically stressed conditions, like pesticides or synthetic fertilizers, may reflect reduced ecological resilience and disrupted nutrient cycling [57]. The structure observed in the FH0.5 network suggests that heat treatment may alter the functional properties of frass in a way that limits beneficial biological interactions.

In our study, although traditional nematode indices showed little variability among treatments and over time, network analysis revealed profound changes in community connectivity, modularity, and complexity. This contrast likely results from differences in ecological scale and sensitivity between these approaches. Indices aggregate broad functional traits and may capture periods of community stability or recovery [37], while network metrics detect alterations in co-occurrence and trophic relationships, which indicate reorganization of the soil food web in response to frass amendment [7]. Functional redundancy among nematode taxa may also underline stability of indices despite dynamic species replacement or interactions [58]. Thus, the combination of both analytical paradigms yields a more complete picture of nematode community responses that includes compositional stability and the complexity of interactions underlying ecosystem resilience.

As far as the nematicidal activity is concerned on the phytoparasitic nematodes *M. incognita*, it seems that *T. molitor* frass exhibits significant paralysis activity on nematode J2s. Most interestingly, the crude frass was significantly more effective than the sanitized frass throughout the entire duration of the experiment. The higher activity observed in the crude frass treatment may be related to its chemical composition and/or to oxygen depletion caused by the larger active microbial biomass in the immersion wells at the start of the assay. Thus, this study contributes to the emerging literature on insect frass by exploring the nematicidal activity of *T. molitor* frass on *M. incognita* assessed in paralysis bioassays. In the same frame, Kisaakye et al. [59] proved that black soldier fly frass extracts suppressed *M. incognita* on spinach, while Wang et al. [60] proved that for Eupolyphaga (*Eupolyphaga sinensis* Walker) frass and its extracts on *M. inchbognita*. Nonetheless, to date, the effect of heat treatment has never been previously studied on the nematicidal activity of frass. In this frame, we are currently studying the diversity of frass chemical composition under the sanitation procedure and its impact on biological activity.

In summary, the structural differences observed in nematode networks underscore the potential of organic amendments like frass to support functionally balanced and ecologically stable soil communities, reinforcing their value in sustainable soil management. This finding together with the first indication of nematicidal activity of frass on plant parasitic nematodes *M. incognita* strengthens the role of frass as a plant protectant and soil improver.

## 5. Conclusions

In conclusion, FH0.5 emerged as the most effective treatment, supporting a nematode community characterized by high functional diversity, a balanced trophic structure, and reduced dominance of plant-parasitic taxa. This amendment favored the activity of enrichment-opportunistic bacterivores and contributed to the gradual re-establishment of a structured and ecologically stable soil food web. The observed patterns indicate that nematode communities initially respond to organic inputs with shifts in composition and network connectivity, followed by signs of recovery and adaptation over time. Most importantly, *T. molitor* frass exhibited significant nematicidal activity on *M. incognita*, especially in its crude form, that is without sanitizing at 70 °C. These findings underscore the potential of carefully selected organic amendments to enhance soil biological quality and support ecosystem resilience. From a sustainable agriculture perspective, the use of processed organic by-products such as frass, particularly in low doses, may offer a viable strategy to improve soil health while minimizing ecological disturbance.

## Figures and Tables

**Table 1:** Classification of nematode genera identified in this study, including their colonizer–persister (cp) values and trophic group assignments.

Genera	C-p Value	Trophic Group
*Acrobeles*	2	Bacterivore
*Acrobeloides*	2	Bacterivore
*Aphelenchoides*	2	Fungivore
*Aphelenchus*	2	Fungivore
*Bitylenchus*	3	Ectoparasitic Herbivore
*Helicotylenchus*	3	Semi-Endoparasitic Herbivore
*Chiloplacus*	2	Bacterivore
*Diploscapter*	1	Bacterivore
*Dorydorela*	4	Omnivore
*Eucephalobus*	2	Bacterivore
*Heterocephalobus*	2	Bacterivore
*Panagrolaimus*	1	Bacterivore
*Plectus*	2	Bacterivore
*Rhabditis*	1	Bacterivore
*Mesorhabditis*	1	Bacterivore
*Meloidogyne*	3	Sedentary endoparasitic Herbivore
*Monhystera*	2	Bacterivore

**Table 2:** List of network metrics that showed the most significant differences among the treatments after 40 DAA (Days After Application) of C: control; FH0.5: heated frass in 0.5% dose; FH1: heated frass in 1% dose; F0.5: raw frass in 0.5% dose; F1: raw frass in 1% dose; MFP: *M. azedarach* fruit powder.

Co-Occurrence Network Metrics					
Treatment	Nodes	Edges	Average Degree	Density	Average Clustering
C	13	24	3.692	0.308	0.531
FH0.5	13	21	3.231	0.269	0.456
FH1	13	28	4.308	0.359	0.486
F0.5	14	40	5.714	0.44	0.533
F1	14	39	5.571	0.429	0.7
MFP	12	31	5.167	0.47	0.605

**Table 3:** Mean values (±standard error) for the Maturity Index (MI), Enrichment Index (EI), Channel Index (CI), Structure Index (SI), and Plant Parasitic Index (PPI) under various treatments at two sampling times, 5 and 40 days after application (DAA). Treatments include control (C), heated frass at 0.5% (FH0.5) and 1% (FH1) doses, raw frass at 0.5% (F0.5) and 1% (F1) doses, and *Melia azedarach* fruit powder (MFP). Identical letters above columns denote mean values that do not differ significantly (two-way ANOVA with Tukey’s post hoc test; * *p* < 0.05; ** *p* < 0.01; ns: not significant; *n* = 4 for all cases). No adjustments for multiple comparisons were applied.

		MI	EI	SI	CI	PPI
5DAA	C	1.85 ± 0.09 abc	49.53 ± 9.87 a	5.33 ± 5.33 a	21.87 ± 8.37 bcd	1.00 ± 1.00 a
	FH0.5	1.89 ± 0.03 abc	43.82 ± 7.02 ab	3.51 ± 3.51 a	29.77 ± 5.12 b	2.00 ± 1.00 a
	FH1	1.79 ± 0.04 bc	59.51 ± 3.70 a	3.60 ± 3.60 a	21.40 ± 2.35 bcd	2.00 ± 1.00 a
	F0.5	1.91 ± 0.10 abc	45.51 ± 6.10 ab	9.52 ± 9.52 a	23.84 ± 11.67 bc	3.00 ± 0.00 a
	F1	1.79 ± 0.04 bc	59.10 ± 4.32 a	6.34 ± 3.51 a	14.40 ± 1.24 bcd	2.00 ± 1.00 a
	MFP	1.87 ± 0.04 abc	45.99 ± 3.41 ab	2.84 ± 2.84 a	21.27 ± 7.30 bcd	3.00 ± 0.00 a
40DAA	C	2.00 ± 0.02 a	21.78 ± 1.07 bc	2.42 ± 2.42 a	77.31 ± 9.35 a	3.00 ± 0.00 a
	FH0.5	1.96 ± 0.01 ab	19.17 ± 2.64 c	0.00 ± 0.00 a	32.03 ± 1.31 b	3.00 ± 0.00 a
	FH1	1.75 ± 0.11 c	56.19 ± 14.12 a	1.42 ± 1.42 a	13.82 ± 8.80 bcd	2.00 ± 1.00 a
	F0.5	1.88 ± 0.06 abc	43.02 ± 10.03 abc	9.36 ± 1.73 a	10.28 ± 3.11 bd	3.00 ± 0.00 a
	F1	1.75 ± 0.10 c	54.36 ± 17.68 a	6.16 ± 3.10 a	5.09 ± 4.60 d	3.00 ± 0.00 a
	MFP	1.79 ± 0.02 bc	54.91 ± 1.62 a	2.67 ± 2.67 a	4.40 ± 1.36 d	2.00 ± 1.00 a
	D	ns	ns	ns	*	ns
	T	**	**	ns	**	ns
	D × T	ns	ns	ns	**	ns

**Table 4:** Different insects’ breeding frass paralysis effect on *Meloidogyne incognita* second-stage juveniles (J2) after 24, 48, and 72 h of immersion in test solutions.

Insect name	**24 h**			**48 h**			**72 h**		
	EC_50_ (% *v*/*v*)	SE	95% CI	EC_50_ (% *v*/*v*)	SE	95% CI	EC_50_ (% *v*/*v*)	SE	95% CI
*Tenebrio molitor*	10.66	0.47	9.09–11.05	10.66	0.47	9.10–11.15	10.04	0.92	8.12–11.96
*Tenebrio molitor heated*	13.84	0.29	13.23–14.44	20.85	0.32	20.18–21.51	20.66	0.46	19.69–21.63

SE: Standard error; CI: Confidence interval.

## Data Availability

The original contributions presented in this study are included in the article/Supplementary Material. Further inquiries can be directed to the corresponding author(s).

## Supplementary Materials

The following supporting information can be downloaded at: https://www.mdpi.com/article/10.3390/biotech14030071/s1. Table S1 (a,b): Simpler complementary table indicating the number of genera that were dominant among the treatments at 5 (1st table) and 40 (2nd table) days after frass application (DAA). (C: control; FH0.5: heated frass in 0.5% dose; FH1: heated frass in 1% dose; F0.5: raw frass in 0.5% dose; F1: raw frass in 1% dose; and MFP: *M. azedarach* fruit powder).
