# Supplementary material for: Dual Role of Tenebrio molitor Frass in Sustainable Agriculture: Effects on Free-Living Nematodes and Suppression of Meloidogyne incognita
Source: BioTech (Basel). 2025 Sep 8;14(3):71. doi: 10.3390/biotech14030071 (PMC12452486; doi:10.3390/biotech14030071)
Supplement: Supplementary file 1 [file biotech-14-00071-s001.zip › biotech-3780946-supplementary.pdf]

Table S1 (a,b). Simpler complementary table indicating the number of genera that were dominant among the treatments at 5(1st table) and 40 (2nd table) days after Frass application(DAA).(C: Control; FH0.5: Heated Frass in 0.5% dose; FH1: Heated Frass in 1% dose; F0.5: Raw Frass in 0.5% dose; F1: Raw Frass in 1% dose; and MFP: M. azedarach fruit Powder).

**(a)**	**FH1**		**F0.5**		**F1**		**C**		**MFP**	
	Helicotylenchus	32.27	*Helicotylenchus*	32.89	*Eucephalobus*	22.62	*Helicotylenchus*	38.03	*Helicotylenchus*	45.69
	Acrobeloides	47.97	*Acrobeloides*	56.44	*Panagrolaimus*	40.94	*Eucephalobus*	53.58	*Acrobeloides*	60.09
**FH0.5**	*Aphelenchus*	60.44	*Eucephalobus*	66.52	*Helicotylenchus*	59.26	*Acrobeloides*	66.28	*Aphelenchus*	66.51
	Panagrolaimus	71.23	*Mesorhabditis*	71.36	*Acrobeloides*	71.45	*Panagrolaimus*	74.81	*Bitylenchus*	70.76
	**22.07**		**26.77**		**27.94**		**31.48**		**21.46**	
			Helicotylenchus	26.97	*Eucephalobus*	26.79	*Helicotylenchus*	37.66	*Helicotylenchus*	35.65
			Acrobeloides	51.26	*Helicotylenchus*	42.92	*Acrobeloides*	52.64	*Acrobeloides*	54.1
	**FH1**		*Eucephalobus*	60.99	*Acrobeloides*	58.42	*Eucephalobus*	64.67	*Aphelenchus*	65.89
			Aphelenchus	69.8	*Panagrolaimus*	73.02	*Panagrolaimus*	73.79	*Panagrolaimus*	74.53
			Panagrolaimus	77.12						
			**29.91**		**28.86**		**34.67**		**25.03**	
					Helicotylenchus	22.6	*Helicotylenchus*	43.79	*Helicotylenchus*	33.67
					Eucephalobus	43.1	*Acrobeloides*	57.34	*Acrobeloides*	48.09
			**F0.5**		*Panagrolaimus*	58.82	*Eucephalobus*	69.75	*Eucephalobus*	59.73
					Aphelenchus	71.41	*Panagrolaimus*	76	*Aphelenchus*	65.55
									Mesorhabditis	70.94
					**26.51**		**38.4**		**22.67**	
							Helicotylenchus	29.6	*Helicotylenchus*	24.76
							Eucephalobus	49.81	*Eucephalobus*	47.24
					**F1**		*Panagrolaimus*	62.51	*Panagrolaimus*	65.23
							Acrobeloides	72.09	*Aphelenchus*	76.55
							**34.48**		**26.54**	
									Helicotylenchus	46.81
									Eucephalobus	59.12
							**MFP**		Acrobeloides	68.89
									Panagrolaimus	75.28
									**36.44**	

(b)	FH1		F0.5		F1		C		MFP	
	Acrobeloides	49.84	*Acrobeloides*	46.86	*Acrobeloides*	44.09	*Acrobeloides*	58.36	*Acrobeloides*	46.25
	Diploscapter	64.95	*Panagrolaimus*	55.78	*Diploscapter*	57.45	*Helicotylenchus*	77.15	*Mesorhabditis*	58.95
**FH0.5**	*Helicotylenchus*	77.43	*Helicotylenchus*	63.74	*Eucephalobus*	69.4			*Heterocephalobus*	66.3
			Diploscapter	70.94	*Rhabditis*	81.15			*Panagrolaimus*	73.23
	**42.54**		**38.53**		**59.4**		**35.36**		**58.0797**	
			Acrobeloides	58.91	*Acrobeloides*	52.3	*Acrobeloides*	33.12	*Acrobeloides*	53.99
			Diploscapter	67.45	*Eucephalobus*	63.29	*Diploscapter*	54.81	*Mesorhabditis*	66.27
	**FH1**		*Panagrolaimus*	74.19	*Rhabditis*	73.56	*Aphelenchus*	67.07	*Heterocephalobus*	72.75
							Rhabditis	76.33		
			**51.71**		**67.63**		**36.06**		**67.87**	
					Acrobeloides	38.15	*Acrobeloides*	60.17	*Acrobeloides*	36.77
					Diploscapter	53.85	*Panagrolaimus*	67.88	*Mesorhabditis*	55.2
			**F0.5**		*Rhabditis*	68.47	*Diploscapter*	74.04	*Heterocephalobus*	62.63
					Eucephalobus	81.96			*Diploscapter*	68.43
									Eucephalobus	73.83
					**36.4**		**51.82**		**36.82**	
							Acrobeloides	50.17	*Acrobeloides*	24.37
							Diploscapter	62.26	*Mesorhabditis*	37.84
					**F1**		*Rhabditis*	72.89	*Rhabditis*	50.93
									Diploscapter	64
									Eucephalobus	75.46
							**72.13**		**33.19**	
									Acrobeloides	53.82
							**C**		*Mesorhabditis*	66.19
									Heterocephalobus	72.93
									**69.78**	
